# Candidate Gene Identification of Feed Efficiency and Coat Color Traits in a C57BL/6J × Kunming F2 Mice Population Using Genome-Wide Association Study

**DOI:** 10.1155/2017/7132941

**Published:** 2017-07-30

**Authors:** Yuanxin Miao, Fathia Soudy, Zhong Xu, Mingxing Liao, Shuhong Zhao, Xinyun Li

**Affiliations:** ^1^Key Laboratory of Agricultural Animal Genetics, Breeding and Reproduction of Ministry of Education and Key Laboratory of Swine Genetics and Breeding of Ministry of Agriculture, Huazhong Agricultural University, Wuhan 430070, China; ^2^The Cooperative Innovation Center for Sustainable Pig Production, Wuhan 430070, China; ^3^Jingchu University of Technology, Jingmen 448000, China; ^4^Jingmen Animal Husbandry and Veterinary Bureau, Jingmen 448000, China

## Abstract

Feed efficiency (FE) is a very important trait in livestock industry. Identification of the candidate genes could be of benefit for the improvement of FE trait. Mouse is used as the model for many studies in mammals. In this study, the candidate genes related to FE and coat color were identified using C57BL/6J (C57) × Kunming (KM) F2 mouse population. GWAS results showed that 61 and 2 SNPs were genome-wise suggestive significantly associated with feed conversion ratio (FCR) and feed intake (FI) traits, respectively. Moreover, the Erbin, Msrb2, Ptf1a, and Fgf10 were considered as the candidate genes of FE. The Lpl was considered as the candidate gene of FI. Further, the coat color trait was studied. KM mice are white and C57 ones are black. The GWAS results showed that the most significant SNP was located at chromosome 7, and the closely linked gene was Tyr. Therefore, our study offered useful target genes related to FE in mice; these genes may play similar roles in FE of livestock. Also, we identified the major gene of coat color in mice, which would be useful for better understanding of natural mutation of the coat color in mice.

## 1. Introduction

Feed efficiency (FE) is an important trait in livestock. In pigs, feed takes more than 60% of the total costs. Improvement of FE is one of the most important approaches for reducing the productive cost of livestock. At present, the assessment of FE is by using two highly correlated indicators: FCR (feed conversion ratio) and RFI (residual feed intake) [1, 2], which lower FCR, and RFI means better FE [3–5].

Mice were widely used as animal model in energy metabolism, feed intake, and FE studies [6–8]. Recent study showed that oxygen consumption was negatively associated with FE of mice. Also, the higher oxygen consumption mice have higher mitochondrial activity and energy expenditure than lower oxygen consumption mice [9, 10]. These results indicated that energy metabolism was negatively related to FE in mice. Similarly, the energy metabolism pathway in the skeletal muscle tissue was found negatively related to FE in pigs [11, 12]. Further, lots of studies confirmed that the hypothalamus can regulate the food intake of mice [13–15]. The* Grelin*,* Leptin*,* MC4R*,* NPY,* and* Agrp* genes have been confirmed to participate in the regulation of feed intake in mice through hypothalamus neurons [16–18]. Orally administered tributyltin (TBT) can significantly increase the FE through inhibiting feed intake in mice [19]. In pigs, feed intake was also negatively related to FE and the* MC4R* has been confirmed as a candidate gene of FE in pigs [20–22]. These studies indicate that mice are the ideal model for FE study of livestock.

The molecular mechanisms of the FE trait of farm animals have been partially revealed in previous studies. It has been reported that genes involved in lipogenic and steroidogenic pathways were downregulated in the liver and fat tissues of high FE pigs. Further, signaling pathway analysis indicated that* ESR1* could affect the FE trait via regulating acute caloric restriction of pigs [23]. Our former study showed that vitamin A metabolism pathway in liver tissue played important roles in regulation of FE in pigs [24]. Furthermore, FE trait could also be affected by immune response [25]. The genes that were expressed in hypothalamus and participated into lipid metabolism were also found to regulate FE in Angus cattle and chicks [26, 27].

Some candidate genes related to FE have been identified in previous studies. One study showed that* GLP1R*,* CDKAL,* and* SGMS1*genes, which related to insulin release, were associated with pig RFI and ADFI traits based on the GWAS results of 1,400 pigs [28]. Also,* MAP3K5*,* PEX7,* and* DSCAM* gene were suggested as candidate genes of RFI traits based on the GWAS results of 1,272 pigs [29]. In addition,* HIF1AN* on SSC4 and* LBX1* on SSC14 have been speculated as possible candidate genes of FCR based on the GWAS results of 3,071 Duroc pigs [30]. Though some candidate genes of FE have been identified, few of them can be mutually authenticated in different studies, and the major genes of FE were still largely unknown.

In this study, the candidate genes related to FE have been identified using mouse model. According to the GWAS results,* Erbin*,* Msrb2*,* Ptf1a,* and* Fgf10* genes closely linked with the top 3 significant SNPs were considered as candidate genes of FCR. The* Lpl* genes closely linked with the 2 genome-wise suggestive significant SNPs of FI was considered as its candidate gene. Also, the* Tyr* gene was suggested as the major genes of coat color of mice.

## 2. Results

### 2.1. FCR and ADG Are High Correlated in C57 × KM F2 Population

The growth rate and FE traits were of significant difference between KM and C57 mice. KM mice growth was much faster and they have better FE compared to C57 mice at the age of 3 to 5 weeks. Therefore, a C57 × KM F2 segregation population has been constructed and the AFI, FCR, and ADG traits at 3 to 5 weeks of each of the F2 mice were measured. As a result, ADG of F2 mice is 0.74 g/day, AFI of F2 mice is 4.32 g/day, and FCR is 6.04. Statistic analysis indicated that the ADG, FI, and FCR traits were significantly different between males and females (Table 1). Male mice have better FE and higher AFI and growth rate than those of female mice. Correlation analysis indicated there were high correlations between ADG with final body weight (FBW) (*R* = 0.77), FI (*R* = 0.65), and FCR (*R* = −0.6), while there was weak correlation between FI and FCR (*R* = 0.11) (Table 2).

### 2.2. The Results of RAD-Seq and SNP Calling

After the F2 population construction, 34 extremely high FCR and 38 extremely low FCR were selected for RAD-Seq. In the high FE group, FCR of females is 8.33 ± 1.59; and FCR of males is 7.81 ± 1.45. In the low FE group, FCR of females is 4.85 ± 0.42, and FCR of males is 4.76 ± 0.53 (Table 3). For the RAD-Seq, more than 500 million clean reads were yielded. The ratio of clean data in raw data were greater than 90%, the error rates were less than 0.05%, the Q20 and Q30 were greater than 90%, and the GC contents were close to 40% (Table 4). After sequence, the SNPs were identified. As a result, 988,717 raw SNPs were firstly identified according to RAD-Seq.

### 2.3. GWAS Results of FCR and AFI Traits

**Figure 1:**
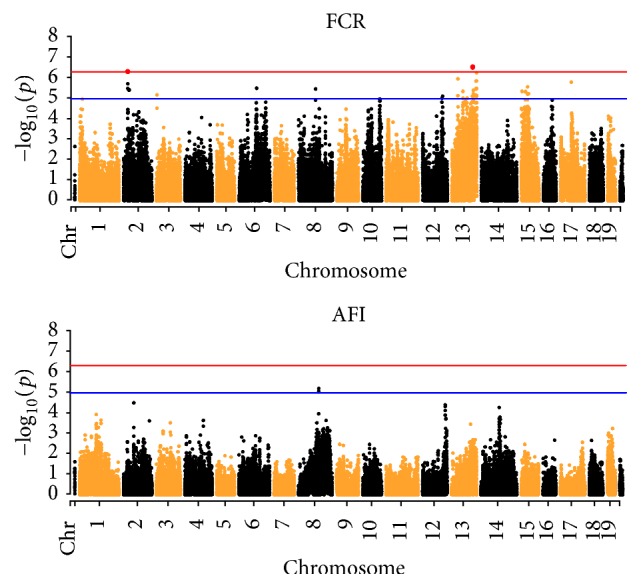
Manhattan plot of genome-wide association analysis studies for FCR and AFI. The red and blue lines indicate Bonferroni corrected* P* = 0.05 and 1, respectively.

In order to identify the candidate genes and SNPs of FE trait, GWAS was performed by using 92132 high quality SNPs. As a result, 61 SNPs associated with FCR traits were found at genome-wise suggestive significant level (*P* < 1). Among them, 2 SNPs were significantly associated with FCR traits (*P* < 0.05), located at chromosomes 13 and 2, respectively (Figure 1). The Erbin, Msrb2, Ptf1a, and Fgf10 genes were closely linked with the top 3 SNPs of FCR (Table 5). Also, 2 SNPs associated with AFI were found at genome-wise suggestive significance (*P* < 1) (Figure 1), which was closely linked with Lpl gene (Table 5).

### 2.4. GWAS Result Indicated That Tyr Gene Was the Candidate Gene of Coat Color

**Figure 2:**
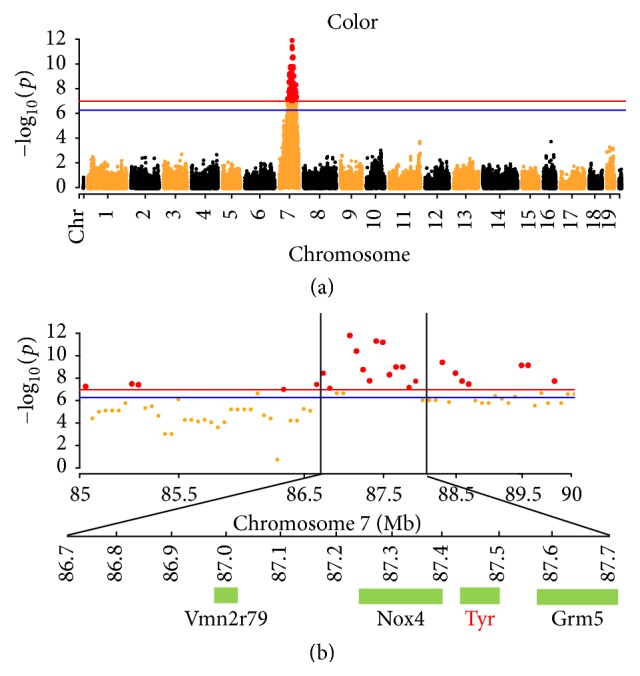
Manhattan plot and the candidate genes of coat color traits. The red and blue lines indicate Bonferroni corrected* P* = 0.01 and 0.05, respectively. Four genes were closely linked with the 13 most significant SNPs.

The coat color of KM mice is white and that of C57 mice is black. In the F2 population, there were 459 black and 181 white ones. The ratio of black : white is approximately 3 : 1. The 72 mice were chosen for RAD-Sequencing, including 49 black and 23 white. The GWAS result showed there was strong signal on chromosome 7 (Figure 2). In total, 332 SNPs were significantly associated with the coat color at genome-wide level (*P* < 0.05) (Figure 2(a)). In the 87.16~87.68 Mb, there were 13 significant SNPs (*P* < 0.01). The most significant SNP was closely linked with Vmn2r79,* Nox4*,* Tyr,* and* Grm5 *genes (Figure 2).

## 3. Discussion

Feed efficiency is an important economic trait in farm animals. Identification of the major genes would be useful for improving this trait. At present, although some signaling pathways have been suggested to be related to FE, not many candidate genes have been suggested. Moreover, few candidate genes have been validated. One possible reason is that FE is a complex economic trait, which may be regulated by many genes. The effect of each gene may not be very large. The second reason is that the population used for FE study may not be good enough. The sample size may not be big enough due to restriction of funding or labor. At present, the major genes of FCR were still not very clear. In this study, we adopt a new strategy. The two mouse strains with significant difference at FE traits were chosen, and the extremely high FE and low FE F2 mice were chosen for GWAS analysis. Some candidate genes of FE have been identified using the F2 mice population. As we know, mice are used as a model for many studies due to high conservation of genome between mammals. Therefore, these candidate genes could also be useful for the improvement of FE in farm animals.

In this study, four candidate genes of FCR have been identified. Functional study showed that* Erbin *could bind and segregate phosphorylated* Smad2/3* complex, which blocked the TGF-*β* signaling [31, 32]. Also, the phosphorylation of* Smad2/3* can transduce signals of MSTN, one of the most powerful inhibitors of muscle growth [33–35]. Moreover,* Erbin* has been reported to inhibit cardiac hypertrophy via* ERK* signaling pathway [36, 37]. Therefore, we deduce that Erbin may regulate FE trait through affecting muscle growth especially via TGF- *β* signaling pathway. Msrb2 is highly expressed in skeletal muscle tissue, which is an oxidoreductase in the mitochondria and responsible for elimination of intracellular reactive oxygen species (ROS) [38, 39]. Many studies indicated that ROS play important roles in muscle growth and increase ROS level accompanied with muscle mass loss [40–42]. Therefore, we deduced that* Msrb2* may regulate FE through affecting the ROS level in the skeletal muscle tissue.* Ptf1a* gene is the key factor for the fate determination of the pancreatic exocrine cells which is indispensable for pancreas development [43].* Ptf1a*-null mice were dead soon after birth, and inhibition of* Ptf1a* resulted in pancreatic hypoplasia, glucose intolerance, and insufficient insulin secretion in a dosage-dependent manner [44].* Fgf10* has been reported to participate in adipogenesis.* Fgf10* knock-out mice die shortly after birth, and the preadipocyte proliferation and adipogenesis are greatly impaired. Signaling pathway studies indicated that* FGF10* could activate FGF receptor 2b (*FGFR2b*) and stimulates preadipocyte proliferation and adipogenesis through the downstream Ras/MAPK/C/EBP*α* pathway [45]. Glucose and adipose metabolism are also important for the regulation of FE [12]. Therefore,* Ptf1a* and* FGF10* gene may participate into regulation of FE through affection glucose and adipose metabolism.

For feed intake trait, Lipoprotein lipase* (Lpl)* was suggested as the candidate gene in this study. Previous studies indicated that* Lpl* is a multifunctional enzyme that plays major roles in the metabolism and transport of lipids in peripheral tissues [46, 47]. Muscle-specific* Lpl* transgenic mice showed TG accumulation and insulin resistance [48, 49]. Also, muscle-specific* Lpl* knock-out could increase the insulin mediated glucose uptake in the skeletal muscle tissue [50].* Lpl* also presents in brain; specific mice with knock-out of* Lpl* in neuron (NEXLPL−/−) are hyperphagic and obesity. Functional study showed that AgRp and Mc3r were significantly upregulated in the neuron specific* Lpl* knock-out mice [51]. These studies indicated that* Lpl* played important roles in energy metabolism, which could affect feed intake, glucose, and lipids metabolism in mice. Thus, we conclude that* Lpl* may be candidate gene of AFI of mice.

The candidate gene for the coat color has also been analyzed. The genotypes of the mice have been detected using the top significant SNP. The heterozygosity/homozygosity ratios in the high FE group (AA : AC : CC = 5 : 18 : 11) and low FE group (AA : AC : CC = 5 : 20 : 13) have no significant difference. This indicated that the coat color trait was not correlated with the FE trait. According to the GWAS results, the* Tyr* gene has been considered as the major gene of coat color trait.* Tyr* is known to be the rate-limiting enzyme affecting the production of melanin pigment [52], which oxidates tyrosine to dihydroxyphenylalanine (DOPA) and determines which type of melanin could be synthesized [53]. Previous studies have shown that mutations of the* Tyr* gene are associated with albinism phenotype of mouse [54], human [55], cattle [56], and rat [57]. Therefore, we conclude* Tyr* is the major gene of coat color trait.

## 4. Materials and Methods

### 4.1. Population Constructed

In this study, a F2 segregation population of C57BL/6 (C57) × Kunming (KM) mice was constructed. C57 mice are black, which grow slow and have low FE. KM mice are white, which grow fast and have high FE. Thus, 7 KM females and 7 C57 males were chosen as the founder for F2 population construction. In total, 640 F2 mice including 319 females and 321 males were generated. All mice were housed under controlled temperature (21 ± 2°C) on a 12 : 12 hour light-dark cycle with free access to food and water. The body weight at 3 weeks and 5 weeks and the total feed intake of this period of each of the F2 mice were measured. Furthermore, average day gain (ADG), average day feed intake (AFI), and feed conversion ratio (FCR) were measured. All the methods in this study were carried out in accordance with the approved guidelines from the Regulation of the Standing Committee of Hubei People's Congress. Furthermore, all experimental protocols were approved by the Ethics Committee of Huazhong Agricultural University (HZAUMU2013-0005).

### 4.2. DNA Preparation and RAD-Sequencing

The tail samples of the F2 mice were collected at 5 weeks of age. Total DNA of mouse tail was extracted with E-Z 96® Mag-Bind® Tissue DNA kit (omega, USA), according to the manufacturer's instructions. Gel electrophoresis and NanoDrop ND2000 spectrophotometry (Thermo Fisher Scientific, USA) were used to detect the quality and concentration of DNA. Then 34 extremely high and 38 extremely low FCR F2 mice were chosen for RAD-Sequencing. RAD-Sequencing was performed by a commercial company service (Novogene, Beijing, China). In brief, mice DNA samples were firstly digested by using EcoR I restriction enzymes. Then, the P1 and P2 adapters were ligated onto the DNA fragments. The P1 and P2 adaptor contains the Illumina PCR Forward and Reverse primer sequences. After 12-cycle PCR amplification, the 350~550 bp DNA fragments were collected and sequenced by using Illumina HiSeq2000.

### 4.3. SNP Mining and GWAS Analysis

The reads quality of the raw data was first evaluated and the low quality reads were filtered according to the criteria (*N* > 10%;* Q* <= 5). Then, the adapter sequence of the high quality reads were trimmed, which were named as clean reads. The clean reads were then mapped to mouse genome (Version number GRCm38.75) using BWA (Burrows-Wheeler Aligner) software. The PCR redundancy was removed by using Picard-tools. The unique mapping reads with less than 3 mismatch Nt were chosen for SNP calling. The SNPs were called by using GATK software under the criteria (Qual score ≥ 30, QD < 20.0, ReadPosRankSum < −8.0, FS > 10.0 and QUAL < $MEANQUA). Those SNPs with the criteria calling rate ≥ 70%, MAF ≥ 1%, and HWE* P* ≥ 10^−6^ were identified. Subsequently, the missing SNPs were amputated by using Beagle Genetic Analysis software. GWAS analysis was finally performed using the 92132 SNPs and 72 samples with the criteria MAF ≥ 5%, genotyping call rate ≥ 95%, and HWE* P* ≥ 10^−6^. The significant SNPs were analyzed by using Plink software based on case/control model. Bonferroni corrected* P *values were adopted for the genome-wide significance threshold that was set as 1/*N*, 0.05/*N,* and 0.01/*N,* three levels, where* N* is the number of total SNPs used for GWAS.

### 4.4. Statistic Analysis

The differences of the traits between male and female mice were analyzed by analyses of variance using SAS. Pearson's correlation coefficients of difference in traits were counted by using SAS.

## 5. Conclusions

In conclusion, we constructed a C57 × KM F2 segregation population. The results of GWAS indicated that the* Erbin*,* Msrb2*,* Ptf1a,* and* Fgf10* genes could be candidate genes of FCR in mice.* Lpl* could be candidate gene of AFI in mice. These genes may also be the candidate genes of the FCR and AFI traits in livestock. Also,* Tyr* is considered as the candidate gene of coat color in mice. This study offered new candidate genes for FE, which would be useful for bettered understanding of the mechanisms of FE trait. Also, the major gene of coat color offered new evidence of genetic variation of this trait in mice.

## Figures and Tables

**Table 1:** The descriptive statistics of growth traits of F2 generation mice.

	F2	F2	
		♀	♂
Number	**640**	**319**	**321**
The initial body weight (IBW, g)	10.26 ± 1.88	10.19 ± 1.87	10.33 ± 1.90
The final body weight (FBW, g)	20.62 ± 2.97	19.62 ± 2.54^A^	21.62 ± 7.34^B^
Average daily gain (ADG, g)	0.74 ± 0.17	0.67 ± 0.14^A^	0.81 ± 0.16^B^
Average feed intake (AFI, g)	4.32 ± 0.75	4.08 ± 0.66^A^	4.56 ± 0.76^B^
Feed conversion ratio (FCR)	6.04 ± 1.47	6.24 ± 1.27^A^	5.84 ± 1.63^B^

^A,B^Different letters within a row represent significant differences at *P* < 0.01.

**Table 2:** The correlation analysis between the traits of F2 generation mice.

	IBW	FBW	ADG	FI	FCR
IBW	1.00				
FBW	0.63	1.00			
ADG	−0.01	0.77	1.00		
AFI	0.33	0.72	0.65	1.00	
FCR	0.27	−0.30	−0.60	0.11	1.00

**Table 3:** The descriptive statistics of growth traits for RAD-Seq individuals.

	High FCR		Low FCR	
	♀ (*n* = 17)	♂ (*n* = 17)	♀ (*n* = 21)	♂ (*n* = 17)
IBW (g)	9.51 ± 1.67	11.19 ± 1.52	8.53 ± 1.55	9.03 ± 2.00
FBW (g)	24.73 ± 10.50	25.53 ± 6.79	24.05 ± 7.35	22.88 ± 4.57
ADG (g)	0.52 ± 0.15	0.61 ± 0.19	0.80 ± 0.13	0.91 ± 0.15
AFI (g)	4.16 ± 0.87	4.59 ± 1.06	3.85 ± 0.56	4.31 ± 0.64
FCR	8.33 ± 1.59	7.81 ± 1.45	4.85 ± 0.42	4.76 ± 0.53

**Table 4:** The sequence quality status of RAD-Seq data.

	High FCR	Low FCR
Raw base (10^8^ bp)	18.79 ± 14.54	13.13 ± 5.76
Clean base (10^8^ bp)	17.67 ± 13.81	12.28 ± 5.56
Effective rate (%)	93.62 ± 1.80	93.04 ± 1.72
Error rate (%)	0.03 ± 0.00	0.03 ± 0.00
Q20 (%)	96.88 ± 0.29	96.61 ± 0.58
Q30 (%)	91.46 ± 0.53	90.78 ± 1.36
GC content (%)	40.21 ± 0.62	40.48 ± 0.82

Effective rate (%): the percentage of clean data in raw data; error rate (%): base error rate; Q20 and Q30 (%): the percentage of base with Phred value for greater than 20 or 30; GC content (%): the percentage of G and C base.

**Table 5:** The candidate genes in the regions of 0.5 Mb nearby the suggestive significant SNPs for FCR and AFI.

Trait	CHR	SNP location	*P *value	Adjacent genes (±0.5 Mb)
FCR	13	103458907	3.36*E* − 07	*Erbin*
	2	19827406	5.40*E* − 07	*Msrb2 Ptf1a*
	13	118659655	6.23*E* − 07	*Fgf10*

AFI	8	69019203	6.82*E* − 06	*Lpl *
	8	69019179	9.76*E* − 06	*Lpl *
